# Prevalence and Clinical Relevance of T-Helper Cells, Th17 and Th1, in Hepatitis B Virus-Related Hepatocellular Carcinoma

**DOI:** 10.1371/journal.pone.0096080

**Published:** 2014-05-27

**Authors:** Jian Yan, Xiao-Long Liu, Gang Xiao, Ning-Lei Li, Yi-Nan Deng, Lu-Zhe Han, Liang-Chun Yin, Li-Juan Ling, Li-Xin Liu

**Affiliations:** 1 Department of General Surgery, The Third Affiliated Hospital, Southern Medical University, Guangzhou, China; 2 Guangdong Provincial Key Laboratory of Liver Disease Research, Guangzhou, China; 3 Department of Hepatic Surgery, The Third Affiliated Hospital of Sun Yat-sen University, Guangzhou, China; Harvard Medical School, United States of America

## Abstract

**Background and Aims:**

An immune imbalance in the cytokine profile exerts a profound influence on the progression of hepatitis B virus (HBV) infections and hepatocellular carcinoma (HCC). The present study evaluated the immune status of T helper (Th) 17 and Th1 cells in patients with HBV-related and non-HBV-related HCC.

**Methods:**

We randomly enrolled 150 patients with HCC. Blood samples and tissue samples were obtained. The distributions and phenotypic features of Th17 and Th1 cells were determined by flow cytometry and/or immunohistochemistry.

**Results:**

Compared to corresponding non-tumor regions, the levels of Th17 and Th1 cells were significantly increased in tumors of patients with HCC (P<0.001). The intratumoral densities of IL-17-producing cells and IFN-γ-producing cells were associated with overall survival (OS, P = 0.001) and disease-free survival (DFS, P = 0.001) of patients with HCC. The ratio of Th17 to Th1 in HBV-related HCC was higher than in non-HBV-related HCC. A multivariate Cox analysis revealed that the Th17 to Th1 ratio was an independent prognostic factor for OS (HR = 2.651, P = 0.007) and DFS (HR = 2.456, P = 0.002).

**Conclusions:**

HBV infections can lead to an imbalance in immune status in patients with HCC. An elevated Th17 to Th1 ratio may promote tumor progression. The Th17 to Th1 ratio could serve as a potential prognostic marker for scoring the severity of HCC.

## Introduction

Hepatocellular carcinoma (HCC) is the fifth most common cancer, and its incidence is increasing worldwide, due to the dissemination of Hepatitis B virus (HBV) infections [1], particularly in China. Despite recent advances in treatments, such as surgery, ablation, interventional treatments, and liver transplantation, the most important treatment for HCC is surgical resection. However, tumor recurrence and metastasis remain the major obstacles for long-term survival, and overcoming these obstacles has attracted increasing attention. The overall recurrence rate attributable to distant metastasis or intrahepatic reappearance can be as high as 65% or 43%, respectively, after treatment [2]. This poor outcome has been attributed to the highly vascular nature of HCC, which increases its propensity for spreading and invading neighboring or distant tissues [3]–[5].

Many studies have indicated that inflammation may play a critical role in the development of cancer. In fact, about 15% of the global cancer burden is attributable to infectious agents [6]. Moreover, increased risk of malignancy is associated with chronic inflammation caused by chemical and physical factors [7]. The inflammatory microenvironment of cancer is characterized by the presence of leucocytes, both in the tumor stroma and within the tumor [8]. Intratumoral lymphocytes may contribute to cancer growth and metastasis, and to the immunosuppression associated with malignant tumors.

Recent studies have demonstrated that CD4+ T cells play an important role in initiating and maintaining antitumor immunological responses. The intratumoral regulatory T cells (Tregs) are associated with HCC invasiveness, and the balance between regulatory and cytotoxic T cells may provide a promising predictor for recurrence and survival in HCC [9]. CD4+ effector T cells can be classified into two groups according to their cytokine profiles. Initially, two different forms of T helper (Th) effectors, type 1 (Th1) and type 2 (Th2), were identified in both mice and humans [10]–[11]. Another type of Th (Th0) cell produces all the cytokines produced by both Th1 and Th2 cells [12]. Th1 cells can produce interferon (IFN)-γ to enhance antimicrobial and antitumor responses; in contrast, Tregs suppress T cell immunity in disease statuses [13]–[15]. Recently, a new Th group, called Th17, was described; this group is different from Th1, Th2, and Th0 cells [16]. Th17 cells produce interleukin 17 (IL-17); they are highly pro-inflammatory, and they induce severe autoimmunity [17]–[21].

It is known that HBV infections are linked to the development of HCC. Some studies have indicated that the percentage of Th17 cells was significantly increased in the peripheral blood of patients with chronic hepatitis B. In contrast, the percentages of Th1 and Tc1 cells were significantly decreased in patients with hepatitis B [22]. However, the prevalence and clinical significance of the ratio of Th17 to Th1 cells in HCC remains unclear. Therefore, in this study, we aimed to determine the Th17 to Th1cellratiosin patients with HBV-related and non- HBV-related HCC. In addition, we determined the clinical significance of IL-17 production by Th17 cells and IFN-γ production by Th1 cells in patients with HCC.

Our results indicated that the levels of Th17 and Th1 cells in peripheral blood were both significantly increased, but this phenomenon was more obvious in patients with HBV-related HCC than in those with non-HBV-related HCC. The densities of intratumoral IL-17-producing cells and IFN-γ-producing cells were associated with overall survival (OS) and disease-free survival (DFS) in patients with HCC. The Th17 to Th1 ratio was higher in patients with HBV-related HCC than in those with non-HBV-related HCC. These results indicated that a HBV infection can lead to an imbalance in the immune status during progression of HCC. This study was the first to propose that the ratio of Th17 to Th1 cells may serve as a potential prognostic marker for scoring the severity of HCC.

## Patients and Methods

### Patients and specimens

Tumor and peripheral blood samples were obtained from 150 patients with HCC, confirmed pathologically at our hospital (Third Affiliated Hospital, Sun Yat-sen University, Guangdong, China), from January 2008 to January 2012. All cases conformed to the diagnostic criteria for HCC according to the American association of studies in liver disease (AASLD). None of the patients received anticancer therapy before sampling. Data were collected on patient demographics, clinical exam findings, and laboratory test results, including patient age, gender, serum alpha-fetoprotein (AFP) level, preoperative imaging data (i.e., abdominal computed tomography [CT] or magnetic resonance [MR] imaging of tumor size, number, and macrovascular invasion), HBV infection, and HBV-DNA level. The final diagnosis of HCC was based on a pathology examination performed after radical resection of the liver cancer. Individuals with a concurrent autoimmune disease were excluded from this study.

No patient had received anticancer therapy before surgery, and no patient demonstrated distant metastasis. Tumor differentiation was graded with the Edmondson grading system. The tumor stage was determined according to the International Union Against Cancer, tumor-node-metastasis (TNM) classification system and the 2010 American Joint Committee on Cancer. This study was approved by the Institutional Review Board of the Key Lab for Liver Disease Research in Guangdong Province. All patients signed an informed consent form.

### Cell isolation

Peripheral blood mononuclear cells (PBMCs) were isolated by Ficoll density gradient centrifugation. Fresh liver tissues were washed, cut into small pieces, and digested with 0.05% collagenase IV and 0.002% DNase I at 37°C for 30 min. Dissociated cells were filtered through a 150-µm mesh.

### Flow cytometric analysis

PBMCs were stained with the following antibodies: fluorescein isothiocyanate (FITC)-conjugated anti-CD4, phycoerythrin-cyanin 7 (PE-Cy7)-conjugated anti-CD4, PE-conjugated anti-IFN-γ (Beckman Coulter, Fullerton, CA, USA), Alexa Fluor 647 (AF647)-conjugated anti-IL-17, allophycocyanin (APC)-conjugated anti-FoxP3, and PE-conjugated anti-Perforin (eBioscience, San Diego, CA, USA). For intracellular cytokine detection, cells were stimulated for 5 h with a leukocyte activation cocktail (BD Biosciences), stained with surface markers, fixed and permeabilized with IntraPre Reagent (Beckman Coulter), and then stained with anti-IL-17 and anti-IFN-γ. Data were acquired with a Cytomics FC500 (Beckman Coulter) and analyzed with CXP analysis software (Beckman Coulter).

### Immunohistochemistry

Formalin-fixed and paraffin-embedded sections (4-µm thick) were cut and mounted on aminopropyltriethoxysilane-treated slides. Slides were routinely prepared by deparaffinizing with xylene and rehydrating with ethanol washes. Nonenzymatic antigen retrieval was performed by microwave heat treatment for 3×7 min in a 0.01 m sodium citrate buffer (pH 6.0). Endogenous peroxidase and nonspecific background staining were blocked by incubating slides with methanol that contained 0.3% H2O2 for 30 min. Slides were washed with phosphate buffered saline (PBS) for 15 min and then incubated with anti-IL-17 and anti- IFN-γ rabbit polyclonal antibodies for 1 h at 37°C. The working dilution for the primary antibodies was 1∶50. Sections were rinsed with PBS for 15 min and then incubated with ENVISION+secondary anti-rabbit antibodies conjugated to horseradish peroxidase for 45 min. After washing with PBS for 15 min, the final products were visualized with the 3-amino-9-ethylcarbazole substrate system. The sections were counterstained with Mayer's hematoxylin for 20 s before mounting. Both positive and negative controls were prepared for each section; we used normal rabbit serum IgG in place of the primary antibody as a negative control. All experiments were performed in duplicate.

### Evaluation of Th17 and Th1 cells

Th17 and Th1 cell data were analyzed by 3 independent observers that were blinded to the clinical outcome. The sections were screened under a microscope with a low power lens (100×). The 10 most representative fields were analyzed at high power (400×) with a Leica DM IRB inverted research microscope. The Th17 and Th1 cell densities were quantified according to the mean number of IL-17-producing and IFN-γ-producing cells, respectively, observed in 10 hot spots (field areas of 0.145 mm^2^).

### Follow-up

After the liver resection treatment, patients were regularly followed up at outpatient clinics. Monitoring was performed with abdominal and chest CT scans every 2 to 3 months during the first two postoperative years, and every 5 to 6 months during the following years. The AFP measurement was performed every month during the first year, and then, every 3 to 6 months for the next 2 to 5 years. In the following years, an annual abdominal CT scan was performed. Suspicious lesions observed on the CT and AFP exams were monitored with CT or MR images acquired of the abdomen, pelvis, chest, and bone. Disease-free survival (DFS) was defined as the period from the date of the hepatectomy to the date that tumor recurrence was detected. Follow-up on all patients was concluded by December 2012. Overall survival (OS) was defined as the period from the date of therapy until death or the end of follow-up.

### Statistical analysis

All statistical analyses were performed with the SPSS 19.0 statistical software package. Patients lost to follow-up were censored at the last follow-up. The t-test was used to analyze independent samples, and Pearson's chi-square (χ2) test was used to analyze differences in clinicopathological features between two groups. A logistic regression model was used to determine independent risk factors. The survival rates after treatment for HCC were analyzed with the Kaplan-Meier survival curve and tested with the log-rank statistic. Preoperative clinicopathological factors that had a significant impact on DFS in the univariate analysis were entered into a multivariate Cox regression model (stepwise forward method) to determine their independent effects. Correlations between variables were determined by linear regression analysis. P-values <0.01 were considered statistically significant.

## Results

### Circulating Th17 and Th1 cells in HBV-related and non-HBV-related HCC

**Figure 1:**
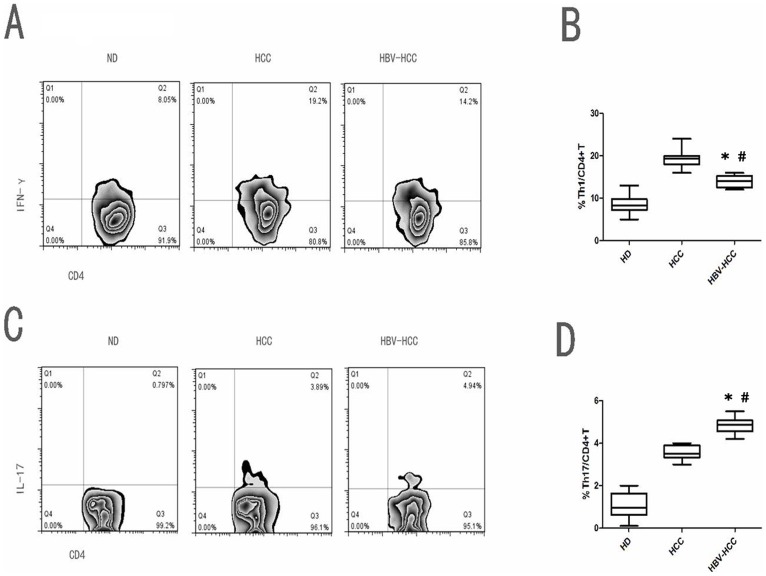
The frequencies of T cell subsets in peripheral blood from patients with HCC and healthy donors. (**A, C**) Representative flow cytometry data, and (**B, D**) statistical analyses compare baseline frequencies of circulating (**A**) IFN-γ-producing CD4+ T (Th1) cells and (**C**) IL-17-producing CD4+T (Th17) cells between patients with HBV-related HCC (HBV-HCC; n = 100), those with non- HBV-related HCC (HCC; n = 50), and healthy controls(ND; n = 50). Th17 cells and Th1 cells were both increased in patients with HCC compared to healthy controls. (**B and D**). Th17 cells were increased in HBV-related HCC more apparently than non- HBV-related HCC; conversely, Th1 cells were increased in non-HBV-related HCC more apparently than HBV-related HCC.

We used flow cytometry to determine the general immune status before and after surgery of 150 patients with HCC (100 with HBV-related HCC and 50 with non-HBV-related HCC) compared to the status of 50 healthy donors. We measured the frequencies of Th1 cells, Th17 cells, and cytokine levels (IL-17, IFN-γ) in peripheral blood collected in the perioperative period. In general, the frequencies of total CD4+ T cells in patients with HBV-related HCC and non-HBV-related HCC did not differ significantly from that of healthy controls. Next, we compared different CD4+ T cell subsets and found that the frequencies of IL-17-producing CD4+T (Th17) cells in patients with HBV-related HCC was higher than in those with non-HBV-related HCC and healthy controls. IFN-γ-producing, CD4+ T (Th1) cells in patients with HBV-related HCC was lower than that in patients with non-HBV-related HCC. Furthermore, the ratio of Th17 to Th1 in patients with HBV-related HCC was higher than that in patients with non-HBV-related HCC and that in healthy controls. These results indicated that an HBV infection may lead to an imbalance in the immune status in patients with HCC. In addition, the cytokines, IL-17 and IFN-γ, may play an important role in the progression of HCC (**Figure 1**).

### Frequency of Th17 and Th1 cells in HBV-related and non-HBV-related HCC

**Figure 2:**
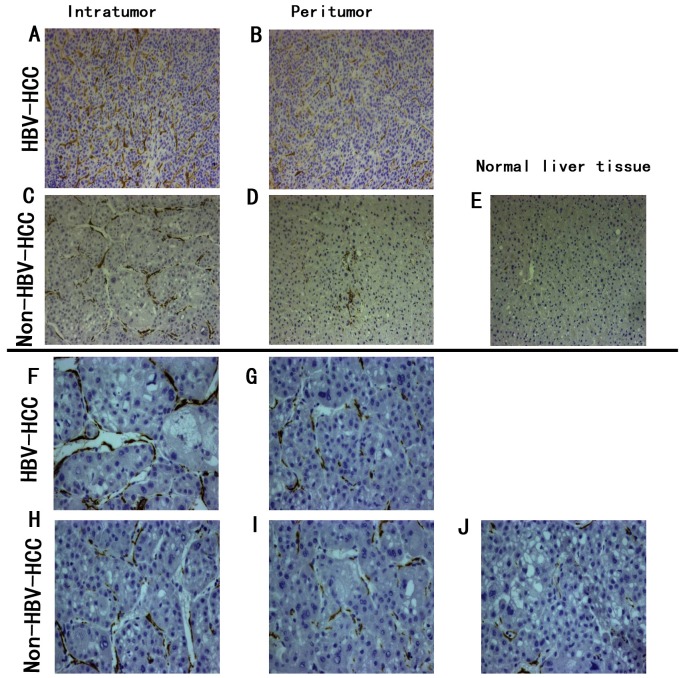
In situ staining shows IL-17 and IFN-γ expression in HBV-related and non- HBV-related HCC tumors. (**A–E**) IL-17-producing cells were stained as diffuse tan or brown particles in immunohistological sections. (**A**)Tissue from HBV-related HCC, and (**B**)paired para-cancerous tissues. (**C**)Tissue from non- HBV-related HCC, and (**D**)paired para-cancerous tissues; (**E**)tissue from normal, healthy liver. (**F–J**)IFN-γ-producing cells were also stained as brown particles in immunohistological sections. (**F**)Tissue from the non-HBV-related HCC, and (**G**)paired para-cancerous tissues; (**H**)tissue from HBV-related HCC, and (**I**)paired para-cancerous tissues; (**J**)tissue from normal, healthy liver.

**Figure 3:**
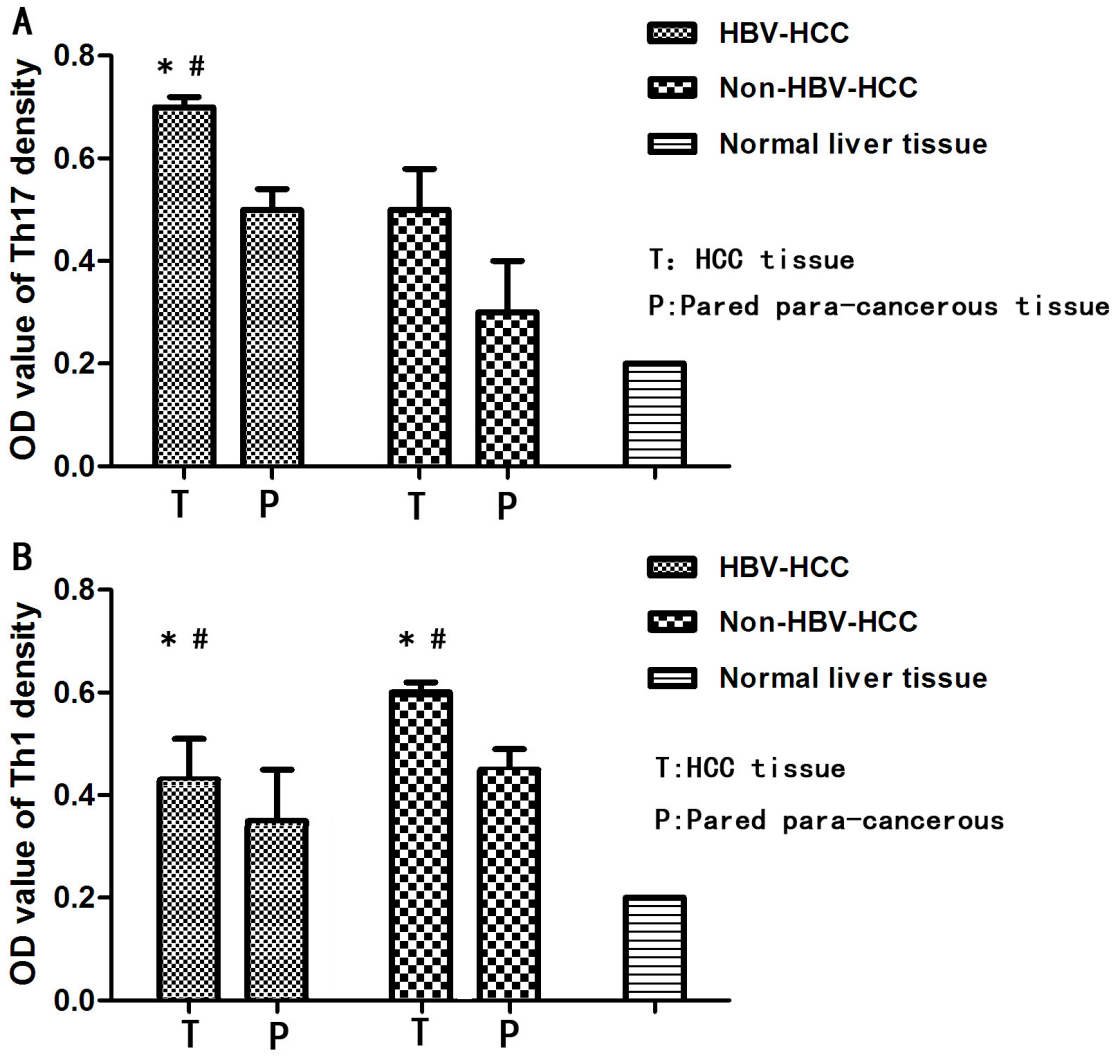
Optical densities (OD) of IL-17-producing (Th17) and IFN-γ-producing (Th1) cells in cancerous and non-cancerous tissues. (**A**)The density of Th17 cells was significantly higher in tumor (T) and paired para-cancerous (P) tissues from patients with HBV-related HCC than in tissues from patients with non- HBV-related HCC. (**B**)The density of Th1 cells was significantly lower in T and P tissues from patients with HBV-related HCC than in tissues from patients with non- HBV-related HCC. (* and # indicate P<0.01).

To evaluate IL-17 and IFN-γ expression in tumors, we performed immunohistochemical staining in paraffin-embedded tissue sections. IL-17-producing and IFN-γ-producing cells were found in the non-tumor, peritumor, and intratumor regions, but they were often most prominent in both the peritumor and intratumor regions. Many Th17 and Th1 cells were present within or surrounding the sinusoids; this finding suggested that some of these cells might migrate between blood vessels and tumor tissues. Compared to normal liver tissue, the levels of Th17 and Th1 cells in cancerous tissues and corresponding adjacent liver tissues were dramatically elevated in HBV-related HCC and non- HBV-related HCC tissues. Moreover, the levels of IL-17-producing and IFN-γ-producing cells in the cancerous tissues of both HCC groups were higher than the levels in the corresponding adjacent liver tissues. We also found that the levels of Th17 and Th1 cells in HBV-related-HCC tissues were higher than in non- HBV-related-HCC tissues (**Figure 2**). The optical density of IL-17 in the HCC and paired para-cancerous tissues was significantly higher in HBV-related HCC tissues than in the non- HBV-related HCC tissues. Conversely, the optical density of IFN-γ in the HCC and paired para-cancerous tissues was significantly lower in HBV-related HCC tissues than in the non- HBV-related HCC tissues (**Figure 3**).

### Th17 and Th1 cell densities in HCC tissues predicted poor survival

**Figure 4:**
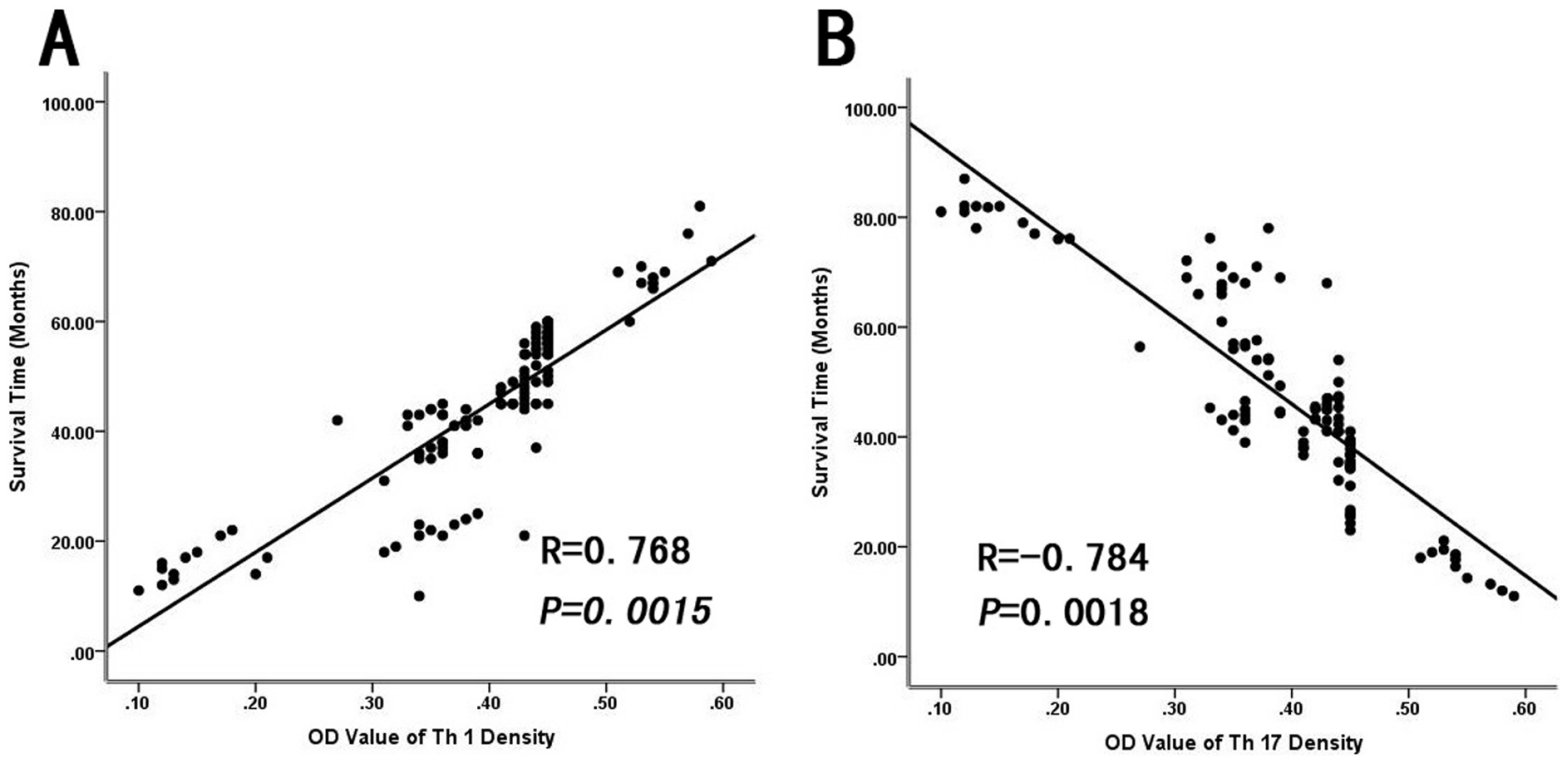
Correlation between levels of IL-17-producing cells, IFN-γ-producing cells, and prognosis for HCC. (**A**)Survival was inversely correlated to the optical density (OD) of intratumoral IL-17-producing cells (R = −0.784, P = 0.0018) and (**B**) positively correlated with the density of IFN-γ-producing cells (R = 0.768, P = 0.0015).

**Figure 5:**
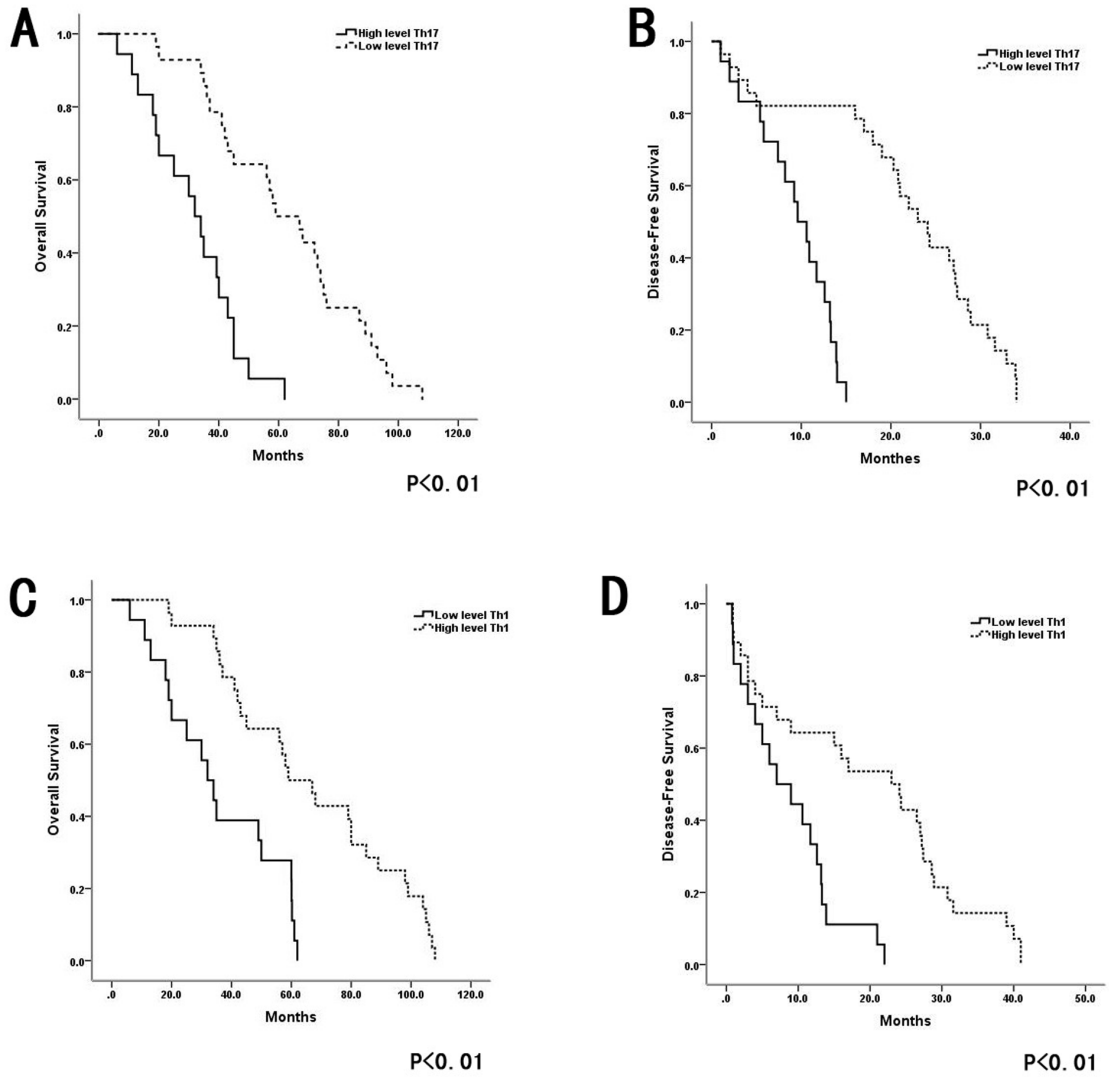
Densities of IL-17-producing cells and IFN-γ-producing cells predicted survival in patients with HCC. (**A and B**)Individuals with high intratumor densities of IL-17- producing cells had significantly shorter OS and DFS than those with low densities. (**C and D**)Patients with high intratumor densities of IFN-γ- producing cells had significantly longer OS and DFS than those with low densities.

To address whether the levels of IL-17-producing cells and IFN-γ-producing cells were associated with HCC progression, we analyzed relevant clinical information and correlated the data with Th17 and Th1 cell densities. As shown in **Figure 4A**, there was a significant inverse correlation between the intratumoral IL-17-producing cell density and patient survival (R = −0.784, P = 0.0018). However, there was a significant positive correlation between the IFN-γ-producing cell density and patient survival (R = 0.768, P = 0.0015; **Figure 4B**). Patients with higher densities of intratumoral IL-17- producing cells had significantly shorter OS (median, 34.1 months) and DFS (median, 7.5 months) than patients with lower densities (medians, OS: 60.1 months; DFS: 24.9 months; P = 0.027 and 0.035, respectively; **Figures 5A, B**). Patients with higher densities of intratumoral IFN-γ-producing cells had significantly longer OS (median, 34 months) and DFS (median, 7 months) than patients with lower densities (medians, OS: 49 months; DFS: 16 months). The peritumoral density of IL-17-producing cells was also associated with OS and DFS (P = 0.039 and 0.005, respectively; **Figures 5C, D**).

### The frequency of circulating Th17 and Th1 cells and the Th17/Th1 ratio in peripheral blood predicted survival

**Figure 6:**
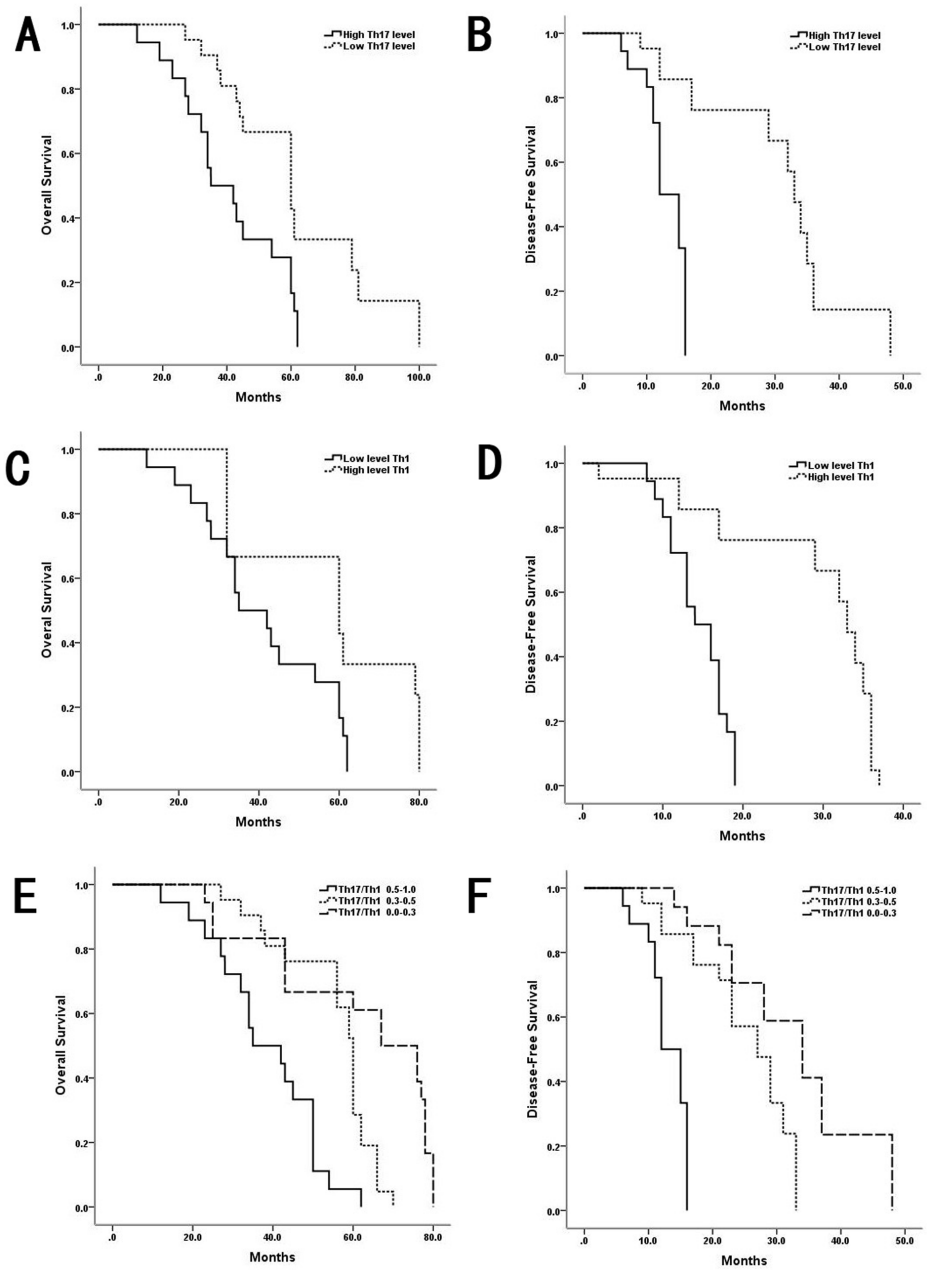
Circulating Th17 and Th1 cells, and their ratio were associated with survival in HCC. (**A and B**)Patients with high levels of circulating Th17 cells had significantly shorter OS and DFS than those with low levels. (**C and D**)Patients with high levels of circulating Th1 cells had significantly longer OS and DFS than those with low levels. (**E and F**)Kaplan-Meier curves reveal a significant difference in the cumulative recurrence and survival rates of patients with different Th17/Th1 ratios. The patients with high Th17/Th1 ratios had significantly shorter OS and DFS than those with low Th17/Th1 ratios.

Because the levels of intratumoral Th17 and Th1 cells were associated with survival in patients with HCC, we next investigated whether circulating levels of Th17 and Th1 cells were associated with survival. We analyzed relevant clinical information and correlated the data with the frequencies of circulating Th17 and Th1 cells and with the Th17/Th1 ratio. Kaplan-Meier analyses revealed that elevated levels of Th17 cells and the Th17/Th1 ratio were negatively associated with OS (P = 0.005) and DFS (P = 0.007). Thus, patients with higher levels of Th17 cells and a higher Th17/Th1 ratio had significantly shorter OS and DFS than those with lower levels and ratios. Similarly, we evaluated the prognostic value of circulating Th1 cells. The results revealed that elevated levels of circulating Th1 cells was significantly positively associated with OS (P = 0.004) and DFS (P = 0.006). A multivariate analysis was used to detect risk factors that affected OS and the recurrence rate (**Table 1**).We analyzed several potential preoperative prognostic predictors of a poor prognosis, including the Child-Pugh score, tumor differentiation, venous invasion, HBV-DNA level>100 IU/ml, elevated Th17 cells, and an elevated Th17/Th1 ratio (**Figure 6**).

**Table 1:** Multivariate analysis of factors that contributed to OS and DFS in patients with HCC.

Variables	P-value of multivariate analysis for OS	HR(95%CI)	P-value of multivariate analysis for DFS	HR(95%CI)
Child-Pugh score	>0.05	NS	>0.05	NS
Tumor differentiation	<0.01	5.47(3.172–7.324)	<0.01	4.373(2.133–5.618)
Intrahepatic vascular invasion	<0.01	4.76(3.145–6.114)	<0.01	3.78(2.198–5.159)
HBV-DNA load	>0.05	NS	>0.05	NS
circulating Th17 cells	<0.01	4.47(2.072–6.324)	<0.01	3.47(2.772–5.314)
circulating Th1 cells	<0.01	5.223(3.071–7.354)	<0.01	5.33(3.132–7.344)
circulating Th17/Th1 ratio	<0.01	4.33(2.172–7.314)	<0.01	4.53(3.162–6.214)

Notes: CI, confidence interval; HR, hazard ratio; NS, not significant.

A Cox multivariate regression analysis was performed for all 7 significant prognostic risk factors. The data revealed that Th17, Th1, the Th17/Th1 ratio, intrahepatic vascular invasion, and tumor differentiation were independent prognostic factors for both OS and DFS (**Table 1**). Additionally, the Cox proportional hazards regression model analysis of these independent prognostic factors revealed that the Wald χ^2^ of the Th17/Th1 ratio was 17.38, higher than that of the other 4 variables (**Table 2**).

**Table 2:** Cox proportional hazards regression model analysis for significant prognostic variables for patients with HCC.

Variables	Waldχ^2^	P	RR	RR(95%CI)
circulating Th17/Th1 ratio	17.38	<0.01	5.46	2.53–18.24
circulating Th17 cells	3.51	<0.01	2.78	1.54–5.34
circulating Th1 cells	4.35	<0.01	2.66	1.74–6.24
Tumor differentiation	5.48	0.043	2.83	1.39–6.24
Intrahepatic vascular invasion	5.65	0.032	2.72	1.89–7.35

Notes: CI, confidence interval; RR, relative risk (hazard ratio).

## Discussion

Some studies have indicated that, although patients with malignancies exhibit a generalized immunologic derangement, an inflammatory reaction at the tumor site can increase tumor growth and progression rates [23]–[26]. Th17 cells can secrete IL-17, IL-21, and IL-22; these cytokines can aggravate the inflammatory status by inducing other inflammatory mediators and leucocytes that gather at inflammation sites [27]–[28].

The present study showed that the levels of Th17 and Th1 cells in peripheral blood were both significantly increased, but this phenomenon was more obvious in patients with HBV-related HCC than in those with non-HBV-related HCC. We found that the intratumoral IL-17-producing cell density was associated with high mortality and reduced survival in patients with HCC. We also found that a high level of Th1 cells was associated with low mortality and extended survival in patients after a HCC resection. Our data have provided new insights into the significance of the pro-inflammatory response in HCC progression.

Th17 cells were previously detected in both murine and human solid tumors [29]–[32], but their contribution to tumor pathogenesis has remained unclear. Some studies in murine systems have shown that IL-17 could impair immune surveillance, and it promoted carcinogenesis and neovascularization in many solid tumors [30], [32]. In contrast, other studies have reported the anti-tumor effect of IL-17 and Th17 cells in mice [33]–[34]. Though the exact underlying mechanism remains unclear, this paradox may be explained by the intensity and nature of IL-17 or IL-17-producing cells. The inflammatory environment driven by relevant levels of endogenous IL-17 may promote tumor progression by stimulating angiogenesis; in contrast, overexpression of IL-17 may lead to inflammatory reactions that trigger cancer-cell destruction [31] and [35].

Some reports have shown that IFN-γ-producing Th1 cells played a key role in anti-tumor immunity [36]. Interestingly, compared to a normal individual, some patients in our study did not have reduced circulating Th1 cell levels, and some even had increased levels (data not shown). One important explanation for that result could be that many HCC samples in our study were derived from chronically inflamed tissues, where inflammation had been induced by a HBV infection, accompanied by a helper T cell response [37]. An alternative explanation could be that the absolute number of Th1 cells did not decrease in patient peripheral blood compared to a healthy person, but the proportion of Th1 cells may have been significantly reduced; therefore, the proportion of Th1 toTh17 cells was imbalanced. In the current study, we observed that the levels of Th17 cells in cancerous tissues were higher than in the corresponding para-cancerous tissues and normal liver tissues. We also found that the levels of Th1 cells in cancerous tissues were lower than in the corresponding para-cancerous tissues and normal liver tissues. The expression of IL-17 in HBV-related HCC was higher than in non-HBV-related HCC tissue. Interestingly, the level of IFN-γ in HBV-related HCC was much lower than in non-HBV-related HCC tissue. Thus, our data strongly suggested that an enrichment inTh17 cells and a relative reduction in Th1 cells inside the tumor may promote progression in patients with HCC. Thus, a chronic HBV infection may lead to an imbalance in the immune status in the body. This notion was supported by our finding that increased intratumoral densities of IL-17-producing cells and relatively decreased densities of Th1 cells were associated with high mortality and reduced survival in patients with HCC. We confirmed that the intratumoral densities of IL-17-producing cells and IFN-γ-producing cells were associated with OS and DFS in patients with HCC. We also found that the Th17 to Th1 ratio was higher in patients with HBV-related HCC than in those with non-HBV-related HCC.

Some studies have indicated that IFN-γ was necessary for cytotoxic effector functions; moreover, it had anti-angiogenic effects in many tumor models [38]. The nature and functional significance of IFN-γ-producing Th1 cells remain unclear, but Th1 cells are found in tissues from patients with autoimmune diseases [39]. The present study showed that a high frequency of IFN-γ-producing cells might be attributed to the fact that most patients with HCC have a chronic HBV infection. This concept was supported by the finding that Th1 cells constituted about 50% of CD4+ cells isolated from either non-tumor or tumor tissues derived from patients with HCC. Thus, we speculate that the tumor promoting and anti-tumor effects of the pro-inflammatory environment driven by these cells may not be static; instead, the salient effects might depend on the timing and context of dynamic changes between Th17 and Th1 cells in the tumor microenvironment.

Recent reports have indicated that both HBV and HCV infections can cause liver exudation, cellular damage, and inflammation. These conditions are closely related to the activation of Th17 cells. In the process of liver disease development and during antiviral treatments, the balance between Th cells in the blood shifts from a predominance of Th1 to a predominance of Th17.This shift indicates a poor prognosis for patients with HCC, because Th17 cells are associated with a high viral load, high transaminase, and activation of mononuclear cells and macrophages in the liver [40]. The HBV protein known as HBcAg is an important antigen that stimulatesTh17 cells. This can lead to elevated expression of CD86, B7H1, CD83 proteins, and the cytokines IL-1β, IL-6, TNF, IL-23p19, and IL-12 in macrophages and monocytes [41]. Other studies have shown that the expression of IL-17 was increased in patients that carried chronic HBV and in patients with cirrhosis, during both the dormant and active periods. IL-17+ cells within liver tissue mainly concentrate in the portal and fibrotic areas. These cells were positively correlated with hyaluronic acid, laminin, and collagen-III, which are known serological indexes of hepatic fibrosis. These findings indicated that lL-17 may be involved in the formation of liver fibrosis. Other studies found that the use of ethylene glycolated interferon plus ribavirin treatment couldlead to are duction inTh17 cells in the liver; this was taken as an indication that liver inflammation was ameliorated [42].

In this prospective study, we have demonstrated that a high frequency of Th17 cells and a low frequency of Th1 cells were associated with a poor prognosis for patients with HCC. We also found that the intratumoral densities of IL-17-producing cells and IFN-γ-producing cells were associated with the OS and DFS in patients with HCC. Furthermore, we demonstrated that the Th17 to Th1 ratio in the peripheral blood of patients with HBV-related HCC was higher than in patients with non-HBV-related HCC. Based on these results, we proposed for the first time that an elevated Th17 to Th1 ratio may promote tumor progression, and that the Th17 to Th1 ratio in peripheral blood could serve as a potential prognostic marker for scoring the severity of HCC.
